# Loss-of-Function Mutations of *BCOR* Are an Independent Marker of Adverse Outcomes in Intensively Treated Patients with Acute Myeloid Leukemia

**DOI:** 10.3390/cancers13092095

**Published:** 2021-04-26

**Authors:** Jan-Niklas Eckardt, Sebastian Stasik, Michael Kramer, Christoph Röllig, Alwin Krämer, Sebastian Scholl, Andreas Hochhaus, Martina Crysandt, Tim H. Brümmendorf, Ralph Naumann, Björn Steffen, Volker Kunzmann, Hermann Einsele, Markus Schaich, Andreas Burchert, Andreas Neubauer, Kerstin Schäfer-Eckart, Christoph Schliemann, Stefan W. Krause, Regina Herbst, Mathias Hänel, Norbert Frickhofen, Richard Noppeney, Ulrich Kaiser, Claudia D. Baldus, Martin Kaufmann, Zdenek Rácil, Uwe Platzbecker, Wolfgang E. Berdel, Jiří Mayer, Hubert Serve, Carsten Müller-Tidow, Gerhard Ehninger, Friedrich Stölzel, Frank Kroschinsky, Johannes Schetelig, Martin Bornhäuser, Christian Thiede, Jan Moritz Middeke

**Affiliations:** 1 Medizinische Klinik und Poliklinik I, Universitätsklinikum Carl Gustav Carus, 01307 Dresden, Germany; Sebastian.stasik@uniklinikum-dresden.de (S.S.); Michael.kramer@uniklinikum-dresden.de (M.K.); Christoph.roellig@uniklinikum-dresden.de (C.R.); g.ehninger@cellex.me (G.E.); friedrich.stoelzel@uniklinikum-dresden.de (F.S.); frank.kroschinsky@uniklinikum-dresden.de (F.K.); Johannes.schetelig@uniklinikum-dresden.de (J.S.); martin.bornhaeuser@uniklinikum-dresden.de (M.B.); christian.thiede@uniklinkum.dresden.de (C.T.); janmoritz.middeke@uniklinikum-dresden.de (J.M.M.); 2 Deutsches Krebsforschungszentrum (DKFZ) and Medizinische Klinik V, Universitätsklinikum Heidelberg, 69120 Heidelberg, Germany; alwin.kraemer@med.uni-heidelberg.de (A.K.);carsten.mueller-tidow@med.uni-heidelberg.de (C.M.-T.); 3 Klinik für Innere Medizin II, Universitätsklinikum Jena, 07740 Jena, Germany; sebastian.scholl@med.uni-jena.de (S.S.); Andreas.Hochhaus@med.uni-jena.de (A.H.); 4 Klinik für Hämatologie, Onkologie, Hämostaseologie und Stammzelltransplantation, Uniklinik RWTH Aachen, 52074 Aachen, Germany; mcrysandt@ukaachen.de (M.C.); TBruemmendorf@ukaachen.de (T.H.B.); 5 Medizinische Klinik III, St. Marien-Krankenhaus Siegen, 57072 Siegen, Germany; r.naumann@mariengesellschaft.de; 6 Medizinische Klinik II, Universitätsklinikum Frankfurt, 60590 Frankfurt am Main, Germany; steffen@em.uni-frankfurt.de (B.S.); serve@em.uni-frankfurt.de (H.S.); 7 Medizinische Klinik und Poliklinik II, Universitätsklinikum Würzburg, 97080 Würzburg, Germany; kunzmann_v@ukw.de (V.K.); einsele_h@klinik.uni-wuerzburg.de (H.E.); 8 Klinik für Hämatologie, Onkologie und Palliativmedizin, Rems-Murr-Klinikum Winnenden, 71364 Winnenden, Germany; Markus.Schaich@rems-murr-kliniken.de; 9 Klinik für Hämatologie, Onkologie, Immunologie, Philipps Universität, 35043 Marburg, Germany; burchert@staff.uni-marburg.de (A.B.); neubauer@staff.uni-marburg.de (A.N.); 10 Klinik für Innere Medizin V, Klinikum Nürnberg Nord, 90419 Nürnberg, Germany; schaefer@klinikum-nuernberg.de; 11 Medizinische Klinik A, Universitätsklinikum Münster, 48149 Münster, Germany; Christoph.Schliemann@ukmuenster.de (C.S.); berdel@uni-muenster.de (W.E.B.); 12 Medizinische Klinik 5, Universitätsklinikum Erlangen, 91054 Erlangen, Germany; stefan.krause@uk-erlangen.de; 13 Medizinische Klinik III, Klinikum Chemnitz, 09116 Chemnitz, Germany; r.herbst@skc.de (R.H.); m.haenel@skc.de (M.H.); 14 Innere Medizin III, HSK Wiesbaden, 65199 Wiesbaden, Germany; norbert.frickhofen@hsk-wiesbaden.de; 15 Klinik für Hämatologie, Universitätsklinikum Essen, 45147 Essen, Germany; richard.noppeney@uk-essen.de; 16 Medizinische Klinik II, St. Bernward Krankenhaus, 31134 Hildesheim, Germany; u.kaiser@bernward-khs.de; 17 Hämatologie und Onkologie, Charité-Universitätsmedizin, 10117 Berlin, Germany; Claudia.Baldus@uksh.de; 18 Abteilung für Hämatologie, Onkologie und Palliativmedizin, Robert-Bosch-Krankenhaus, 70376 Stuttgart, Germany; martin.kaufmann@rbk.de; 19 Department of Internal Medicine, Hematology and Oncology, Masaryk University and University Hospital, 60177 Brno, Czech Republic; racil.zdenek@fnbrno.cz (Z.R.); mayer.jiri@fnbrno.cz (J.M.); 20 Medizinische Klinik und Poliklinik I, Hämatologie und Zelltherapie, Universitätsklinikum Leipzig, 04103 Leipzig, Germany; Uwe.Platzbecker@medizin.uni-leipzig.de; 21 DKMS Clinical Trials Unit, 01309 Dresden, Germany; 22 National Center for Tumor Diseases, 01307 Dresden, Germany

**Keywords:** acute myeloid leukemia, *BCOR*, *BCORL1*, loss-of-function, risk stratification, survival

## Abstract

**Simple Summary:**

Acute myeloid leukemia (AML) is a genetically heterogeneous disease. Clinical phenotypes of frequent mutations and their impact on patient outcome are well established. However, the role of rare mutations often remains elusive. We retrospectively analyzed 1529 newly diagnosed and intensively treated AML patients for mutations of *BCOR* and *BCORL1*. We report a distinct co-mutational pattern that suggests a role in disease progression rather than initiation, especially affecting mechanisms of DNA-methylation. Further, we found loss-of-function mutations of *BCOR* to be independent markers of poor outcomes in multivariable analysis. Therefore, loss-of-function mutations of *BCOR* need to be considered for AML management, as they may influence risk stratification and subsequent treatment allocation.

**Abstract:**

Acute myeloid leukemia (AML) is characterized by recurrent genetic events. The *BCL6* corepressor *(BCOR)* and its homolog, the *BCL6 corepressor-like 1 (BCORL1)*, have been reported to be rare but recurrent mutations in AML. Previously, smaller studies have reported conflicting results regarding impacts on outcomes. Here, we retrospectively analyzed a large cohort of 1529 patients with newly diagnosed and intensively treated AML. *BCOR* and *BCORL1* mutations were found in 71 (4.6%) and 53 patients (3.5%), respectively. Frequently co-mutated genes were *DNTM3A*, *TET2* and *RUNX1*. Mutated *BCORL1* and loss-of-function mutations of *BCOR* were significantly more common in the ELN2017 intermediate-risk group. Patients harboring loss-of-function mutations of *BCOR* had a significantly reduced median event-free survival (HR = 1.464 (95%-Confidence Interval (CI): 1.005–2.134), *p* = 0.047), relapse-free survival (HR = 1.904 (95%-CI: 1.163–3.117), *p* = 0.01), and trend for reduced overall survival (HR = 1.495 (95%-CI: 0.990–2.258), *p* = 0.056) in multivariable analysis. Our study establishes a novel role for loss-of-function mutations of *BCOR* regarding risk stratification in AML, which may influence treatment allocation.

## 1. Introduction

Acute myeloid leukemia (AML) is a genetically heterogeneous disease [1]. In the last decade, next-generation sequencing (NGS) has been introduced in hematological practice, and with the use of myeloid gene panels, rare and recurrent mutations have been unveiled, with a majority of patients harboring more than one mutation even within defined AML entities [2]. The discovery of common recurrent mutations, such as NPM1 and FLT3-ITD, among others, has led to a better understanding of the molecular landscape of AML [1,3], with distinct consequences for prognostication and treatment allocation [4]. However, the role and potential impact of rare mutations on prognosis is not well understood, and warrants further studies to improve risk assessment, implying a more precise approach to AML treatment, as relapse and mortality rates are still unsatisfactory.

The BCL6 corepressor (*BCOR*) gene, and its homolog, the BCL6 corepressor-like 1 (*BCORL1*) gene, are located on chromosomes Xp11.4 and Xq26.1, respectively [5,6]. BCOR was originally described as a repressor of BCL6 [5], but also interacts with PCGF1, KDM2B, MLLT3, and IRF8, while BCORL1 interacts with PCGF1, KDM2B, CTBP1, and HDAC [7]. Both BCOR and BCORL1 are core proteins of the polycomb repressive complex PRC1.1. PRCs are essential for the maintenance of cell identity and cell differentiation, and their perturbance is an important factor in carcinogenesis [8]. PRC1.1 plays a role in epigenetic modification by adding an ubiquitin to histone H2A at lysine 119 [9]. This process (among others) leads to a silencing of Hox gene clusters [10], and mediates transcriptional repression [11]. BCOR is highly expressed in embryonic stem cells, where a role in maintaining the primed pluripotent state has been suggested [7,12]. As a mediator of stemness and differentiation, BCOR is involved in the development of B- and T-cells, as well as erythrocytes [13].

Since BCOR is vital in ectodermal and mesenchymal differentiation, germline loss-of-function mutations in *BCOR* lead to oculofaciocardiodental (OFCD) syndrome in heterozygous females, and prenatal death in hemizygous males [14]. Somatic mutations have been found in a variety of solid tumors, such as retinoblastoma, medulloblastoma, osteosarcoma, and hepatocellular carcinoma [15,16,17,18]. BCOR is an important factor in the regulation of myeloid cell proliferation and differentiation [12]. It has been described in hematologic entities such as aplastic anemia [19,20], chronic myelomonocytic leukemia [21], clonal hematopoiesis of indeterminate potential [22], myelodysplastic syndromes (MDS) [21,23,24,25], and AML [6,21,26,27,28]. In AML, mutations of *BCOR* (m*BCOR*) and *BCORL1* (m*BCORL1*) both occur in 4–6% of patients, and an association with poor outcomes has been suggested [6,26,27,28]. However, given the rarity of these mutations, and the confinement of previous studies to smaller patient cohorts, further investigation of their impact on clinical phenotypes and outcomes in AML seems warranted. We here present the analysis on the impact of BCOR/BCORL1 mutational status on a large cohort of newly diagnosed and intensively treated AML patients.

## 2. Materials and Methods

### 2.1. Data Set

We retrospectively analyzed a multi-center cohort of 1529 AML patients. Eligibility criteria were newly diagnosed AML according to WHO definitions [29], age ≥ 18 years, and available biomaterial at diagnosis. All patients were treated with intensive regimens in the following clinical trials: AML96 [30], AML2003 [31], AML60+ [32], and SORAML [33] or were enrolled in the German Study Alliance Leukemia (SAL)’s AML registry (NCT03188874). Detailed information on treatment regimens is given in the respective references. All mentioned studies were carried out under the auspices of the SAL, and approved by the Institutional Review Board of the Dresden University of Technology (Dresden, Saxony, Germany). All participants gave their written, informed consent, in accordance with to the Declaration of Helsinki.

### 2.2. Definitions

AML was defined as de novo when neither previous malignancy nor previous treatment with chemo- and/or radiotherapy was reported. When myeloid neoplasms were documented prior to AML diagnosis, AML was defined as secondary (sAML). Prior exposure to chemo- and/or radiotherapy before the initial diagnosis defined therapy-associated AML (tMN). Early death was defined as death by any cause within 30 days of the initial diagnosis (ED_30_). Remission and survival criteria were defined according to ELN2017 recommendations [4].

### 2.3. Molecular Analysis

Next generation sequencing (NGS) using a TruSight Myeloid Sequencing Panel (Illumina, San Diego, CA, USA) was performed on pre-treatment bone marrow or peripheral blood, targeting 54 genes (Table S1) associated with myeloid neoplasms, including full coding exons of *BCOR* and *BCORL1,* as previously described in detail [34,35]. A DNeasy blood and tissue kit (Qiagen, Hilden, Germany) was used to extract DNA, and subsequent quantification was performed using a NanoDrop spectrophotometer. Pooled samples were sequenced paired-end (150 bp PE) using a NextSeq NGS instrument (Illumina). The SEQUENCE PILOT software package (JSI medical systems GmbH, Ettenheim, Germany) was used for sequence data alignment, variant calling, and filtering. A 5% variant allele frequency (VAF) cut-off was used. Genome-mapping algorithms were referenced to human genome build HG19. We compared VAFs of *BCOR/BCORL1* mutations with VAFs of co-mutated drivers for dichotomization of dominant, subclonal, and secondary mutations. For putative subclonal mutations a minimum VAF difference of 10% was applied.

### 2.4. Statistical Analysis

We compared categorical variables between groups using the chi-squared test, while continuous variables were compared using the Kruskal–Wallis test. The Kaplan–Meier method was used to estimate survival probabilities. The logrank test was used to compare survival time distributions between groups. Cox regression was used to estimate univariate and adjusted hazard ratios. For the binary endpoint of complete remission, logistic regression models were fitted to estimate univariate and adjusted odds ratios. A significance level of 0.05 was used to determine statistical significance. Calculations were performed in R 4.0.3.

## 3. Results

**Figure 1:**
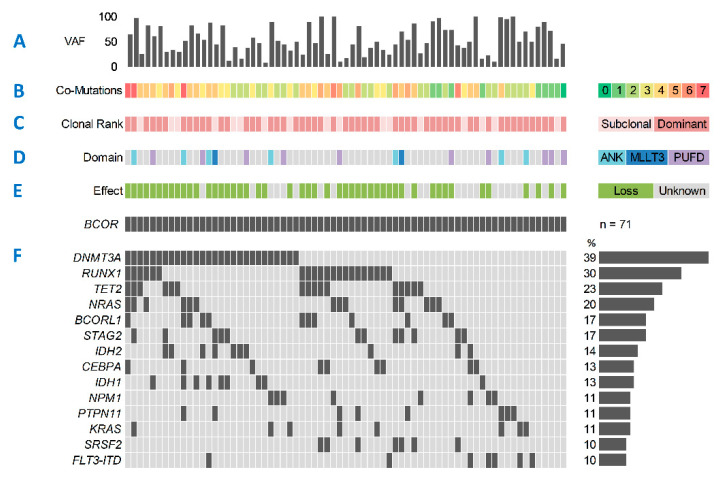
Mutational spectrum of *BCOR*-mutated AML. (**A**) *BCOR* variant allele frequency (VAF) percentages. (**B**) Number of co-mutations in addition to mutated *BCOR*. (**C**) Clonal rank of *BCOR* mutations determined by comparing *BCOR* VAF to co-mutated somatic driver variants. (**D**) Mutated *BCOR* domain, undetermined (grey). (**E**) Effect of mutated *BCOR*, loss-of-function (transcriptional stop, green), or unknown (no transcriptional stop, grey). (**F**) Most common co-mutations. Mutations with a frequency of <10% not shown: *ASXL1, GATA2, WT1, SF3B1, U2AF1, TP53, ZRSR2, IKZF1, KIT, ETV6, EZH2, FLT3-TKD, JAK2, PHF6, CBL, CBLB, CSF3R, NOTCH1, PDGFRA, SMC3*.

**Figure 2:**
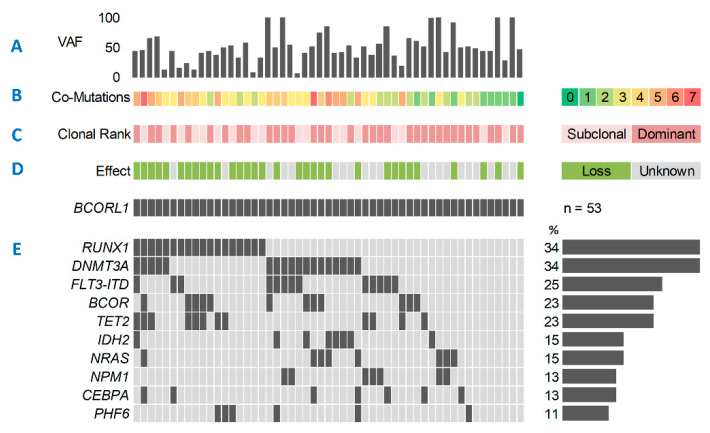
Mutational spectrum of *BCORL1*-mutated AML. (**A**) *BCORL1* variant allele frequency (VAF) percentages. (**B**) Number of co-mutations in addition to mutated *BCORL1*. (**C**) Clonal rank of *BCORL1* mutations determined by comparing *BCORL1* VAF to co-mutated somatic driver variants. (**D**) Effect of mutated *BCORL1*, loss-of-function (transcriptional stop, green), or unknown (no transcriptional stop, grey). (**E**) Most common co-mutations. Mutations with a frequency of <10% not shown: *FLT3-TKD, IDH1, SRSF2, KIT, TP53, PTPN11, SF3B1, U2AF1, WT1, ZRSR2, STAG2, ETV6, EZH2, GATA2, KDM6A, ASXL1, CBL, KRAS, MYD88, SETBP1, JAK2, SMC3.*

In the entire cohort (*n* = 1529), we found m*BCOR* in 71 (4.6%) and m*BCORL1* in 53 (3.5%) patients. Twelve patients (0.8%) concomitantly harbored both m*BCOR* and m*BCORL1*. The median age for the entire cohort was 55 years (Interquartile range (IQR): 44–64). Median variant allele frequency (VAF) for m*BCOR* and m*BCORL1* was 48% (range: 7–100%) and 47% (range: 5–100%), respectively (Figure 1A and Figure 2A). Most patients harboring m*BCOR* (87%) and m*BCORL1* (83%) carried two or more additional mutations in other driver genes, while only one patient in each group had no other co-mutations detected by the panel (Figure 1B and Figure 2B). Both m*BCOR* and m*BCORL1* were more frequently found in the dominant AML clone (76% and 68%, respectively, Figure 1C and Figure 2C). Mutations of *BCOR* were found in several exons and functional domains, including the MLLT3, ANK, and PUFD domains (Figure 1D). The majority (62%) of the mutations were single-nucleotide variants (SNVs). Most mutations had a loss-of-function (LOF) effect (frameshift or non-sense mutations, 68%, Figure 1E), likely resulting in a premature stop of transcription and inactivation of the BCOR protein. The remaining 32% of *BCOR* mutations exclusively comprised missense SNVs, with a presumed damaging effect for the majority of variants, as suggested by the PolyPhen-2 classifier [36].

However, due to the unknown effect of protein function, these mutations were classified as UFs (unknown functions) for sub-analysis. The co-mutational landscape of m*BCOR* AML (Figure 1F) was characterized by high rates of mutation in *DNMT3A* (39%), *RUNX1* (30%), *TET2* (23%), *NRAS* (20%), *BCORL1* (17%), and *STAG2* (17%). Low mutation frequencies were detected for other genes frequently mutated in AML, such as *FLT3* (ITD: 10%, TKD: 3%), *NPM1* (11%), and *TP53* (4%). Similar to m*BCOR*, in m*BCORL1* AML the majority of mutations had a loss-of-function effect (60%, Figure 2D) and were mostly SNVs (62%). The most common co-mutations (Figure 2E) were *DNMT3A* (34%), *RUNX1* (34%), *FLT3-*ITD (25%), *BCOR* (23%), and *TET2* (23%).

Mutations of *BCORL1* were more prevalent in females than in males (m*BCORL1* female 62.3%, male: 37.7%; wild-type *BCORL1* female: 47.2%, male: 52.8%; *p* = 0.044), while for m*BCOR* no such association was observed. For both m*BCOR* and m*BCORL1* no statistically significant associations were detected for age at diagnosis, the presence of a complex karyotype, hemoglobin levels, or platelet counts. With respect to disease status, the rate of sAML was significantly higher amongst patients harboring m*BCOR* than amongst wt*BCOR* patients (21.7% vs. 11%, *p* = 0.023), while no specific association with mutation type (LOF or UF) or m*BCORL1* was found regarding AML type. Outcomes did not differ for patients with sAML and m*BCOR* or m*BCORL1* compared to patients with de novo AML. In the ELN2017 intermediate-risk group we found a higher proportion of LOF of m*BCOR* compared to UF m*BCOR* and wt*BCOR* (LOF m*BCOR*: 86.4% vs. UF m*BCOR*: 28.6% vs. wt*BCOR*: 38.8%, *p* < 0.001), as well as m*BCORL1* compared to wt*BCORL1* (70% vs. 39.1%, *p* < 0.001). Median white blood cell (WBC) counts in LOF m*BCOR* AML were significantly lower compared to UF m*BCOR* and wt*BCOR* (*p* < 0.001), while m*BCORL1* showed no significant association with WBC. Table 1 and Table 2 summarize patient characteristics for m*BCOR* (LOF and UF) and m*BCORL1*, respectively.

**Figure 3:**
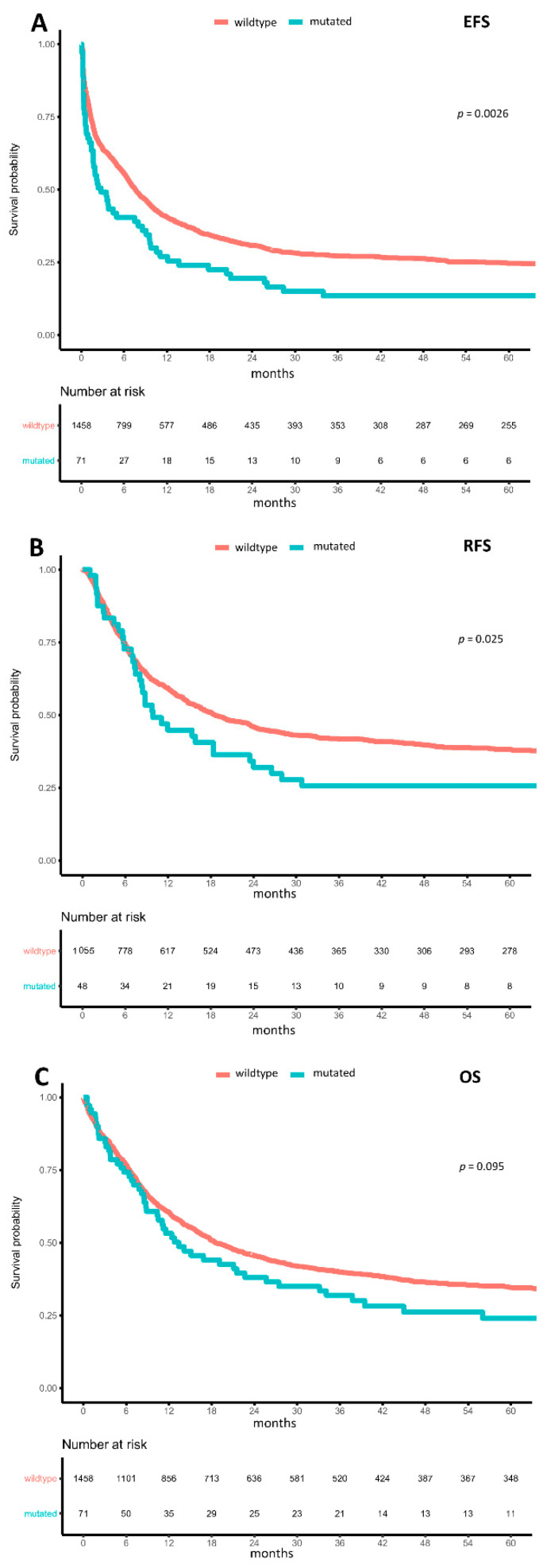
Impact of BCOR mutations on outcomes. Kaplan–Meier plots for (**A**) event-free survival, (**B**) relapse-free survival, and (**C**) overall survival in AML, with wild-type (orange) and mutated (turquoise) BCOR compared using the logrank test.

With respect to clinical outcomes, m*BCOR* and m*BCORL1* were not associated with the rate of complete remission (CR) (m*BCOR*: OR = 0.781 (95%-CI: 0.469–1.301), *p* = 0.342 and m*BCORL1*: OR = 0.795 (95%-CI: 0.442–1.432), *p* = 0.445) or with ED_30_ (m*BCOR*: OR = 0.679 (95%-CI: 0.209–2.199), *p* = 0.518 and m*BCORL1*: OR = 1.288 (95%-CI: 0.454–3.649), *p* = 0.634). In contrast, m*BCOR* was associated with lower median measures of event-free survival (EFS) (2.8 months (95%-CI: 1.7–8.5) vs. 7.6 months (95%-CI: 6.8–8.5), HR = 1.485 (95%-CI: 1.147–1.922), *p* = 0.003; Figure 3A), relapse-free survival (RFS) (9.9 months (95%-CI: 8.1–24.0) vs. 18.7 months (95%-CI: 16.3–23.3), HR = 1.452 (95%-CI: 1.047–2.014), *p* = 0.026; Figure 3B), and overall survival (OS) (13.4 months (95%-CI: 10.4–27.4) vs. 18.5 months (95%-CI: 16.8–21.4), HR = 1.452 (95%-CI: 0.959–1.677), *p* = 0.095; Figure 3C) in a univariate analysis. In a multivariable model adjusted for age, AML type (de novo, sAML, tMN), and ELN2017 risk, m*BCOR* in general was not independently associated with EFS (HR = 1.243 (95%-CI: 0.89–1.736), *p* = 0.202), RFS (HR = 1.407 (95%-CI: 0.93–2.129), *p* = 0.106), and OS (HR = 1.216 (95%-CI: 0.846–1.748), *p* = 0.292).

**Figure 4:**
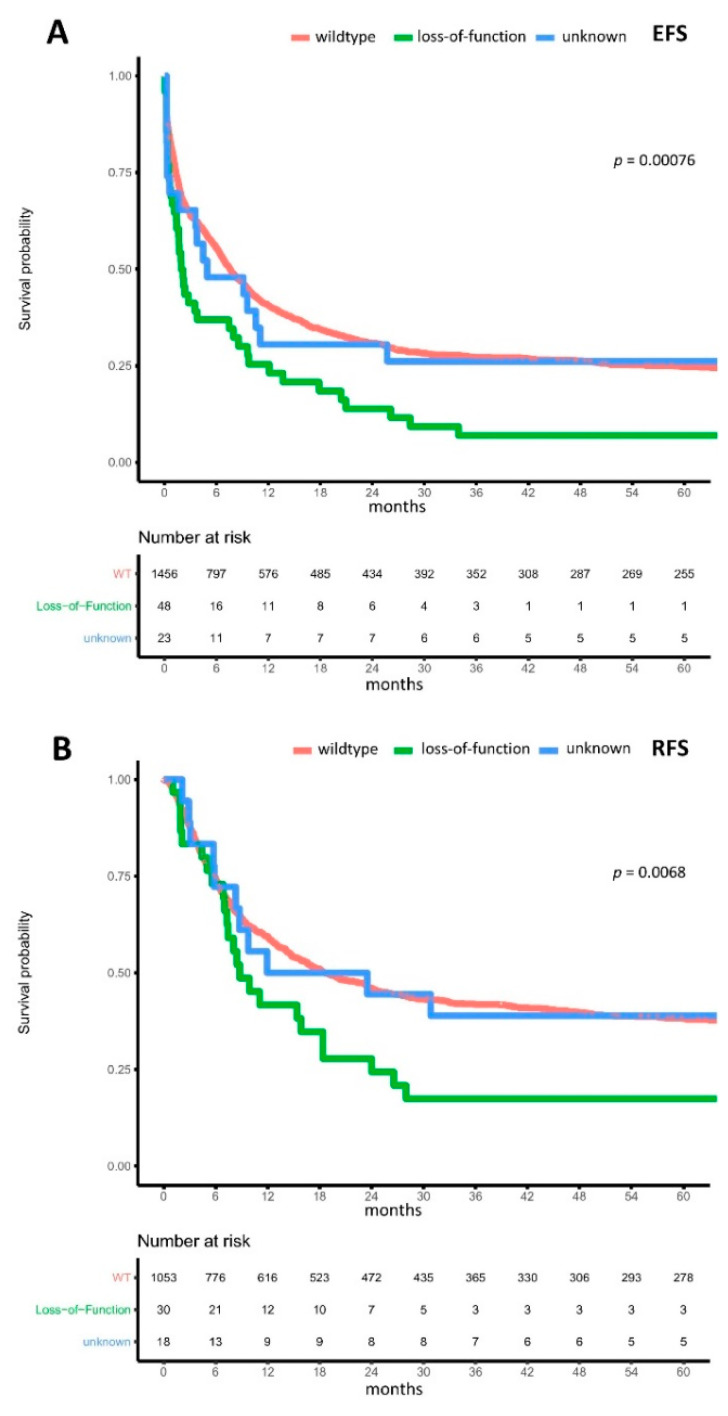

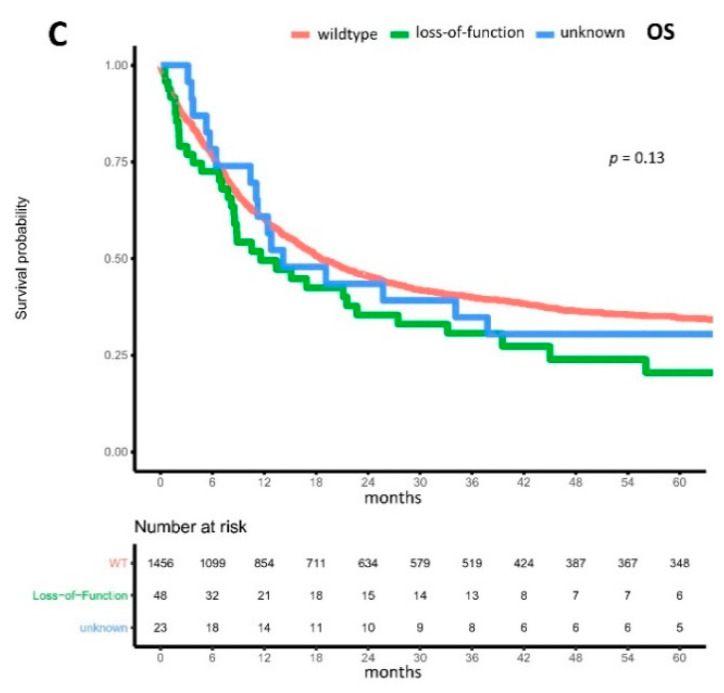
Impact of BCOR loss-of-function mutations on outcomes. Kaplan–Meier plots for (**A**) event-free survival, (**B**) relapse-free survival, and (**C**) overall survival in AML, with mutations of BCOR resulting in a transcriptional stop and loss-of-function (green), BCOR mutations without transcriptional stop resulting in unknown function (blue), and wild-type BCOR (orange) compared using the logrank test.

For patients harboring LOF m*BCOR,* compared to UF m*BCOR* and wt*BCOR,* we found in both univariate and multivariable analysis significantly reduced median EFS (LOF m*BCOR*: 1.9 months (95%-CI: 1.4–8.0) vs. UF variant of m*BCOR*: 4.9 months (95%-CI: 1.676–n.a.) vs. wild-type *BCOR*: 7.5 months (95%-CI: 6.8–8.5), multivariable HR of LOF compared to wt*BCOR* = 1.464 (95%-CI: 1.005–2.134), *p* = 0.047, Figure 4A) and RFS (LOF m*BCOR*: 8.8 months (95%-CI: 7.3–24.0) vs. UF variant of m*BCOR*: 17.7 months (95%-CI: 8.317–n.a.) vs. wild-type *BCOR*: 18.7 months (95%-CI: 16.3–23.3), multivariable HR of LOF compared to wt*BCOR* = 1.904 (95%-CI: 1.163–3.117), *p* = 0.01, Figure 4B). Regarding OS, we found significantly reduced median OS for patients with LOF m*BCOR* variants in univariate analysis (LOF m*BCOR*: 11.6 months (95%-CI: 8.5–33.2) vs. UF variant of m*BCOR*: 14.2 months (95%-CI: 11.045–n.a.) vs. wild-type *BCOR*: 18.4 months (95%-CI: 16.7–21.4), HR of LOF compared to wt*BCOR* = 1.409 (95%-CI: 1.010–1.965), *p* = 0.044), and a strong trend for reduced median OS in a multivariable analysis (HR = 1.495 (95%-CI: 0.990–2.258), *p* = 0.056, Figure 4C). Additionally, adjusting for co-mutations in *DNMT3A*, *TET2,* and *RUNX1* did not significantly affect HR. However, in order to precisely account for multiple interactions, an even larger cohort is needed, due to the rarity of LOF *BCOR*.

**Figure 5:**
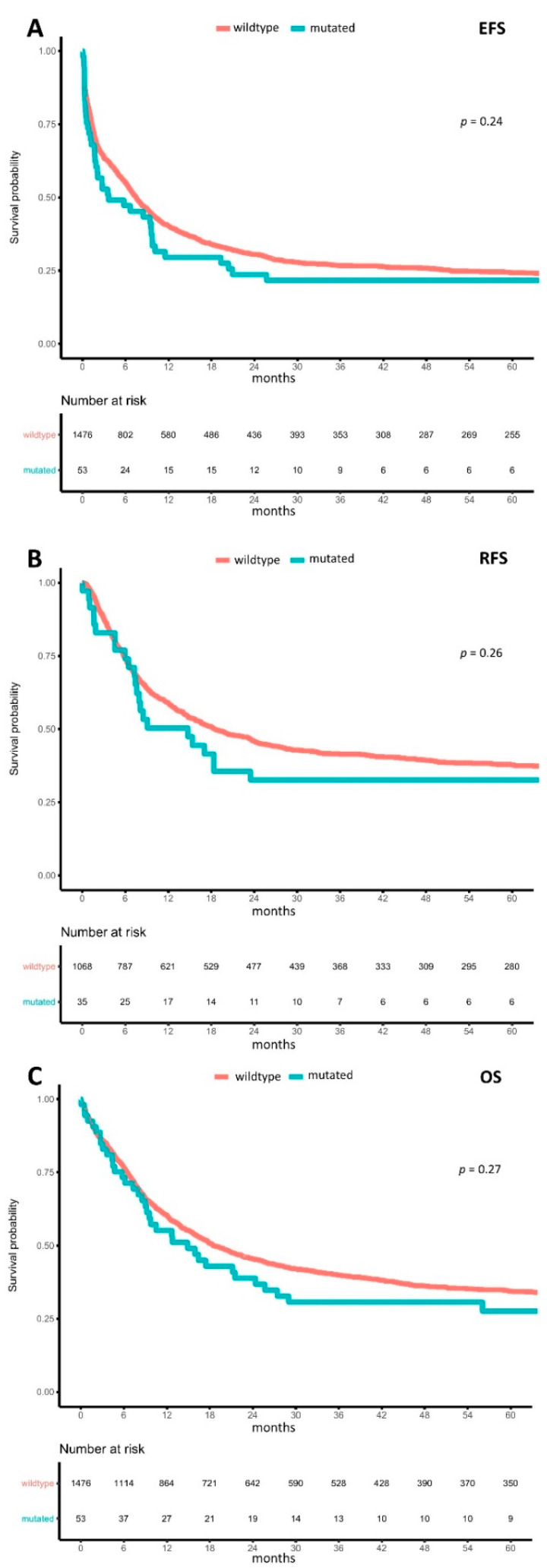
Impact of *BCORL1* mutations on outcome. Kaplan–Meier plots for (**A**) event-free survival, (**B**) relapse-free survival, and (**C**) overall survival in AML, with wild-type (orange) and mutated (turquoise) *BCORL1* compared using the logrank test.

In AML with m*BCORL1* in general compared to wt*BCORL1*, median EFS (3.6 months (95%-CI: 1.9–9.8) vs. 7.5 months (95%-CI: 6.7–8.3), HR = 1.204 (95%-CI: 0.885–1.639), *p* = 0.236; Figure 5A), median RFS (14.8 months (95%-CI 7.6–66.6) vs. 18.5 months (95%-CI: 15.9–23.3), HR = 1.263 (95%-CI: 0.84–1.897), *p* = 0.263; Figure 5B) and median OS (14.9 months (95%-CI: 9.2–27.4) vs. 18.5 months (95%-CI: 16.7–21.4), HR = 1.105 (95%-CI: 0.714–1.71), *p* = 0.654; Figure 5C) were both lower, although none of these differences were statistically significant. Univariate analysis of LOF m*BCORL1* showed significantly reduced EFS (LOF m*BCORL1*: 3.5 months (95%-CI: 1.1–10.1) vs. UF m*BCORL1*: 6.2 months (95%-CI: 1.9–not reached) vs. wt*BCORL1*: 7.5 months (95%-CI: 6.7–8.3), HR = 1.521 (95%-CI: 1.045–2.213), *p* = 0.028); however, in a multivariable analysis adjusting for age, AML type, and ELN2017 risk, this was not statistically significant.

We did not find statistically significant differences in m*BCOR* and m*BCORL1* regarding outcomes in different ELN2017 risk groups. Since few patients with m*BCOR* and m*BCORL1* were in the ELN2017 favorable (*n* = 9 and *n* = 6, respectively) or ELN2017 adverse-risk groups (*n* = 12 and *n* = 6, respectively), we focused on the ELN2017 intermediate-risk group (*n* = 44 and *n* = 35, respectively). In ELN2017 intermediate-risk AML with m*BCOR*, median EFS and OS did not differ compared to wt*BCOR*, while there was a trend of lower median RFS in a univariate model (11.9 months (95%-CI: 7.0–24.0) vs. 14.8 months (95%-CI: 12.5–20.3), HR = 1.446 (95%-CI: 0.977–2.139), *p* = 0.065), as well as in a multivariable model adjusted for age and AML type (HR = 1.637 (95%-CI: 0.995–2.693), *p* = 0.052). In ELN2017 intermediate-risk AML with m*BCORL1*, median EFS and RFS did not differ, but there was a trend of lower median OS in a univariate model (14.9 months (95%-CI: 7.3–24.4) vs. 17.0 months (95%-CI: 14.0–20.6), HR = 1.401 (95%-CI: 0.954–2.058), *p* = 0.086), while in a multivariable model adjusted for age and AML type this difference was not statistically significant. There was no significant association between LOF of m*BCOR* or m*BCORL1* and ELN2017 risk groups regarding outcomes.

## 4. Discussion

We analyzed a large cohort of newly diagnosed and intensively treated AML patients according to their mutational status of *BCOR* and *BCORL1*. The respective proportions of m*BCOR* and m*BCORL1* in the cohort were comparable to those reported in recent studies [6,21,26,27]. We found both m*BCOR* with LOF and m*BCORL1* to be more prevalent in patients in the ELN2017 intermediate-risk group [4]. The proportion of sAML amongst patients harboring m*BCOR* was significantly higher than in their wild-type counterparts, confirming previous reports [37]. Mutations of *BCORL1* were significantly more prevalent in females than in males, as has been previously suggested [27], and patients harboring m*BCOR* with LOF had significantly lower WBC, and a trend for lower peripheral blood blast counts. Mutations of *BCOR* frequently co-occurred with mutations of *DNMT3A, RUNX1, TET2*, *NRAS,* and *BCORL1*. Since both BCOR and DNMT3A function as epigenetic modifiers [7,38], a synergistic role in leukemogenesis has been reported recently [39,40]. In murine models, the co-occurrence of m*BCOR* and mutations of *TET2*, another epigenetic modifier [41] frequently associated with m*BCOR* in our cohort, has been reported to induce MDS [25,42]. This may further reinforce the notion that BCOR’s interaction with other DNA methylators plays a crucial role in leukemogenesis. RUNX1 and BCOR both play essential roles in the proliferation and differentiation of myeloid cells [10,43]. Therefore, the interplay of m*BCOR* and other essential regulators of normal myeloid development appears to be a factor that may promote leukemogenesis. Recent studies suggest, however, that perturbations of BCOR function alone do not suffice to induce malignant transformation [10,21,44], and thus co-mutations appear to be needed in order to drive leukemogenesis or, alternatively, sequential, secondary acquisition of LOF in *BCOR* following an oncogenic event, e.g., MDS driver mutation triggers disease progression towards AML, as suggested by the higher frequency of sAML among m*BCOR* patients. Accordingly, in our cohort of patients with m*BCOR* AML, the majority had at least two co-mutations, while only one patient harbored no other co-mutations targeted by our panel (Figure 1B). Similarly to m*BCOR*, co-mutations of m*BCORL1* were *RUNX1, DNMT3A,* and *TET2*, as well as *BCOR* and *FLT3-*ITD, which were only rarely co-mutations of m*BCOR*. Again, the majority of patients harboring m*BCORL1* had at least two other co-mutations, and only one patient had no other co-mutations revealed by our panel (Figure 2B). As for m*BCOR*, this suggests a potential interplay of impaired BCORL1 function with other dysfunctional mechanisms of DNA methylation, cell differentiation, and signal transduction in leukemogenesis.

Regarding outcomes, we found no statistically significant differences between patients with m*BCOR* and m*BCORL1* regarding CR rate and ED_30,_ compared to wild-type patients. Interestingly, while we observed lower median EFS, RFS, and OS for both m*BCOR* and m*BCORL1* in general, only LOF mutations of m*BCOR* were associated with significantly reduced EFS and RFS and a trend of reduced OS in multivariable testing adjusted for age, AML type, and ELN2017 risk. In MDS, Abuhadra et al. [23] recently reported significantly reduced OS for patients with frameshift mutations of *BCOR*, while general mutation status did not affect OS. Previous studies of m*BCOR* in AML have reported poorer outcomes to often be associated with distinct co-mutations; however, a significant association of LOF mutations of *BCOR* as independent markers of poor outcomes has not yet been reported in AML. Terada et al. [27] reported reduced OS in AML patients who were younger than 65 years, wild-type *FLT3,* and had intermediate-risk cytogenetics. Grossmann et al. [26] reported a higher prevalence of m*BCOR* and a trend of reduced OS in normal-karyotype AML with no mutations in *NPM1*, *FLT3-*ITD, *CEBPA,* or *MLL-PTD*. However, in a validation cohort no association between m*BCOR* and OS was observed. Nevertheless, in both cohorts reduced EFS was detected [26]. Recently, Eisfeld et al. [45] reported reduced survival for patients in the ELN2017 favorable-risk group who harbored mutations in *BCOR* or *SETBP1*. Our findings underline the complexity of the mutational landscape of AML, where even mutational variants of rare mutations have to be considered in order to determine their clinical and prognostic effects. Future work needs to focus on the implementation of LOF mutations of *BCOR* in risk stratification tools for AML management.

## 5. Conclusions

In conclusion, both m*BCOR* and m*BCORL1* are rare but recurrent mutations in AML. While previous studies suggested poor outcomes for m*BCOR* in AML, especially in the context of co-occurring mutations, we found loss-of-function mutations of m*BCOR* to be independent markers of poor outcomes in AML, while m*BCORL1* was not significantly associated with outcomes in multivariable testing.

## Figures and Tables

**Table 1:** Patient characteristics for m*BCOR.*

Parameter	wt*BCOR*	m*BCOR*-LOF	m*BCOR*–UF	*p*-Value
*N*. of patients	1458	48	23	
Age, median (IQR)	55 (44–64)	53 (45–65)	61 (51–69)	0.165
Sex, *n* (%)				0.150
Female	689 (47.3)	26 (54.2)	15 (65.2)	
Male	769 (52.7)	22 (45.8)	8 (34.8)	
AML type, *n* (%)				0.065
De novo	1235 (85.6)	37 (78.7)	15 (68.2)	
sAML	158 (11)	9 (19.1)	6 (27.3)	
tMN	49 (3.4)	1 (2.1)	1 (4.5)	
ELN2017, *n* (%)				<0.001
Favorable	561 (41.7)	1 (2.3)	8 (38.1)	
Intermediate	522 (38.8)	38 (86.4)	6 (28.6)	
Adverse	261 (19.4)	5 (11.4)	7 (33.3)	
Complex karyotype, *n* (%)				0.111
No	1178 (87.8)	44 (93.6)	19 (82.6)	
Yes	164 (12.2)	3 (6.4)	4 (17.4)	
*FLT3*-ITD, *n* (%)				0.022
wt	1114 (76.9)	44 (93.6)	19 (82.6)	
m	334 (23.1)	3 (6.4)	4 (17.4)	
*NPM1*, *n* (%)				<0.001
wt	961 (67)	45 (97.8)	16 (69.6)	
m	474 (33)	1 (2.2)	7 (30.4)	
WBC, median (IQR) in GPt/L	20.85 (4.96–56.4)	4 (1.82–16.88)	16.5 (6.46–34.65)	<0.001
Hb, median (IQR) in mmol/L	5.9 (5.0–7.0)	6.15 (5.25–7.78)	5.59 (5.06–6.01)	0.211
Platelets, median (IQR) in GPt/L	50 (27.0–92.0)	54 (25.5–106)	73 (41.5–118.5)	0.176
LDH, median (IQR) in mmol/L	457 (290.25–795.0)	306 (223.65–458.3)	407 (254.5–897)	0.001
BM blasts (%), median (IQR)	63 (44–79)	58.75 (48.88–72.12)	61 (45–72.73)	0.874
PB blasts (%), median (IQR)	42 (13–75)	19 (6–59)	39 (19.75–70)	0.076

wt: wild-type; m-: mutated; LOF: loss-of-function effect (transcriptional stop); UF: unknown effect (no transcriptional stop); sAML: secondary acute myeloid leukemia; tMN: therapy-related acute myeloid leukemia; ELN2017: European Leukemia Net 2017; WBC: white blood cells; BM: bone marrow; PB: peripheral blood; n/N: number; IQR: interquartile range.

**Table 2:** Patient characteristics for m*BCORL1.*

Parameter	wt*BCORL1*	m*BCORL1*	*p*-Value
N. of patients	1476	53	
Age, median (IQR)	55 (44–65)	52 (43–62)	0.177
Sex,* n* (%)			0.044
Female	697 (47.2)	33 (62.3)	
Male	779 (52.8)	20 (37.7)	
AML type, *n* (%)			0.621
De novo	1244 (85.3)	43 (82.7)	
sAML	167 (11.4)	6 (11.5)	
tMN	48 (3.3)	3 (5.8)	
ELN2017, *n* (%)			<0.001
Favorable	564 (41.5)	6 (12)	
Intermediate	531 (39.1)	35 (70)	
Adverse	264 (19.4)	9 (18)	
Complex karyotype, *n* (%)			0.975
No	1192 (88)	39 (86.7)	
yes	163 (12)	6 (13.3)	
*FLT3*-ITD, *n* (%)			0.782
wt	1138 (77.6)	39 (75)	
m	328 (22.4)	13 (25)	
*NPM1*, *n* (%)			0.006
wt	977 (67.3)	45 (86.5)	
m	475 (32.7)	7 (13.5)	
WBC, median (IQR) in GPt/L	20.09 (4.8–55.27)	14.35 (3.55–54.95)	0.340
Hb, median (IQR) in mmol/L	5.9 (5.0–7.0)	6.2 (5.3–7.0)	0.341
Platelets, median (IQR) in GPt/L	51 (27.0–93.3)	50.5 (30.0–90.5)	0.727
LDH, median (IQR) in mmol/L	452 (288.0–794.8)	333 (254.0–621.0)	0.053
BM blasts (%), median (IQR)	63 (44–79)	65.25 (48.88–82.75)	0.299
PB blasts (%), median (IQR)	40.5 (12–74)	48.5 (15–78)	0.419

wt: wild-type; m-: mutated; sAML: secondary acute myeloid leukemia; tMN: therapy-related acute myeloid leukemia; ELN2017: European Leukemia Net 2017; WBC: white blood cells; BM: bone marrow; PB: peripheral blood; n/N: number; IQR: interquartile range.

## Data Availability

The data presented in this study are available on request from the corresponding author. The data are not publicly available due to privacy issues.

## Supplementary Materials

The following are available online at https://www.mdpi.com/article/10.3390/cancers13092095/s1, Table S1: Summary of the 54 genes targeted by the TruSight Myeloid Sequencing Panel.
