# Supplementary material for: Loss-of-Function Mutations of BCOR Are an Independent Marker of Adverse Outcomes in Intensively Treated Patients with Acute Myeloid Leukemia
Source: Cancers (Basel). 2021 Apr 26;13(9):2095. doi: 10.3390/cancers13092095 (PMC8123716; doi:10.3390/cancers13092095)
Supplement: Supplementary file 1 [file cancers-13-02095-s001.zip › cancers-1169002-supplementary.pdf]

Table S1. Summary of the 54 genes targeted by the TruSight Myeloid Sequencing Panel (Illumina, San Diego, CA, USA).

**TruSight Myeloid Sequencing Panel**				
ABL1	CEBPA	HRAS	MYD88	SF3B1
ASXL1	CSF3R	IDH1	NOTCH1	SMC1A
ATRX	CUX1	IDH2	NPM1	SMC3
BCOR	DNMT3A	IKZF1	NRAS	SRSF2
BCORL1	ETV6/TEL	JAK2	PDGFRA	STAG2
BRAF	EZH2	JAK3	PHF6	TET2
CALR	FBXW7	KDM6A	PTEN	TP53
CBL	FLT3	KIT	PTPN11	U2AF1
CBLB	GATA1	KRAS	RAD21	WT1
CBLC	GATA2	MLL	RUNX1	ZRSR2
CDKN2A	GNAS	MPL	SETBP1	
